# Population study of 1311 C/T polymorphism of Glucose 6 Phosphate Dehydrogenase gene in Pakistan – an analysis of 715 X-chromosomes

**DOI:** 10.1186/1471-2156-10-41

**Published:** 2009-07-30

**Authors:** Bushra Moiz, Amna Nasir, Tariq Moatter, Zulfiqar Ali Naqvi, Mohammad Khurshid

**Affiliations:** 1 Department of Pathology and Microbiology, Aga Khan University, Karachi, Pakistan; 2 Research offices, Aga Khan University, Karachi, Pakistan

## Abstract

**Background:**

Nucleotide 1311 polymorphism at exon 11 of G6PD gene is widely prevalent in various populations of the world. The aim of the study was to evaluate 1311 polymorphism in subjects carrying G6PD Mediterranean gene and in general population living in Pakistan.

**Results:**

Patients already known to be G6PD deficient were tested for 563C-T (G6PD Mediterranean) and 1311 C-T mutation through RFLP based PCR and gene sequencing. A control group not known to be G6PD deficient was tested for 1311C/T only.

C-T transition at nt 1311 was detected in 60/234 X-chromosomes with 563 C-T mutation (gene frequency of 0.26) while in 130 of normal 402 X-chromosomes (gene frequency of 0.32).

**Conclusion:**

We conclude that 1311 T is a frequent polymorphism both in general populations and in subjects with G6PD Mediterranean gene in Pakistan. The prevalence is higher compared to most of the populations of the world. The present study will help in understanding genetic basis of G6PD deficiency in Pakistani population and in developing ancestral links of its various ethnic groups.

## Background

Glucose 6-phosphate dehydrogenase (G6PD) deficiency is one of the most frequent red cell enzymopathies affecting some 400 million people globally[1]. G6PD gene located on human chromosome Xq28 [2], is associated with at least 140 distinct disease producing mutations [3]. These mutations are significant in causing hemolysis, favism, neonatal hyerpbilirubinemia [4]. Besides the disease producing mutations, numerous silent mutations unassociated with any conformational change in enzyme structure have also been demonstrated. Such polymorphisms have greatly helped the geneticists for evaluating clonality of various tumors including uterine myomas [5], lymphomas [6] and other tumors [7]. Further to it, silent mutations of G6PD gene have proved to be invaluable tools in the study of human genome by providing markers for gene mapping [8]and linkage analysis[9]. There is an extensive list of G6PD genetic polymorphism that has been analyzed so far including IVS*5 *at NT 611C-G[10], IVS*2 *NT 97*22*A-G, IVS*11 *NT 93 T-C, IVS*7 *NT 175 C-T and IVS*8 *NT 163 C-T [11]. Unfortunately, many of these polymorphisms had limited utilization as were exclusively observed in African populations.

Of special interest is the polymorphism seen at 1311 nucleotide of exon 11 of G6PD gene[12]. This is widely prevalent in multiethnic non African population of the world and is an important marker in formal genetic analysis of X-linked loci. The nucleotide produces two alleles (1311 C and 1311T) that are observed in both G6PD replete and deficient populations. Polymorphism of 1311 T has been studied extensively in several populations [12,13]. It does not abolish or create any specific site for enzyme restriction and therefore can be detected by creating mismatched antisense primer which produces a site for B*cl*I digestion when 1311T is present [12].

Pakistan with a population of over 165 million people is a home to 18 ethnic groups speaking 60 different languages [14]. The ethnic diversity of this region is attributed to repeated invasions by Aryans [15], Macedonians [16], Arabs and Mongols in its long history [17]. Majority of its people belong to five major ethnic groups: Punjabis, Sindhis, Pathans, Baluch (Balochis) and Mohajirs [18]. Several distinct ethnic groups exist in each major group which has generated complex social, cultural and lingual blend.

The primary objectives were to evaluate NT 1311 polymorphism of G6PD gene in various ethnic groups living in Pakistan and to evaluate its association with 563C-T mutation in G6PD deficient subjects. The secondary objective was to compare the results of 1311 polymorphism with that reported from other populations of the world.

## Methods

The Aga Khan University Hospital Clinical Laboratory uses brilliant cresyl blue dye test (Trinity biotech plc, Wicklow, Ireland) for screening individuals with G6PD deficiency. Subjects reported as G6PD deficient from January 2006 to December 2008 were identified using a computerized institutional database search utilizing International Classification of Disease (v9, American Health Information Management Association, AHIMA, USA). They were contacted via telephone and were requested for participation in the study.

In addition, a control group consisting of healthy subjects who visited the hospital for blood donation was included. These subjects were classified in to various ethnic groups based on subject's self classification. They were verbally scanned through an in-house questionnaire to rule out the possibility of any disorder but were not tested for G6PD deficiency. As the frequency of consanguineous marriages is reported as 31.1% to 58.7% from different places of the country [19,20], some ethnic mix is expected in our cultural groups.

From each participant, 5 ml of blood was collected in an EDTA containing tube afterward white blood cells were separated and stored for DNA extraction according to manufacturer's instructions.

### DNA extraction and PCR/RFLP for screening G6PD 563-T and 1311/T mutations

Genomic DNA was isolated from peripheral blood leucocytes by Wizard^® ^Genomic DNA Purification Kit (Wisconsin, USA). PCR/RFLP technique was used for the detection of G6PD 563C-T and 1311C/T with primers published elsewhere [21]. Each PCR reaction in 25 μl volume contained 34 mM Tris-HCl pH 8.8, 8.3 mM ammonium sulfate, 1.5 mM MgCl_2_, 0.2 mM of each dNTP, 75 ng of each primer, 2 U of *Taq *polymerase (Promega, Wisconsin, USA) and 200 ng of genomic DNA. After 30 cycles consisting of 1 min at 94°C, 30 sec at 58/62°C (respectively for1311C/T and 563 C-T) and 40 sec at 72°C, PCR products were digested overnight at 37°C with either M*bo*II or B*cl*I restriction enzymes. The digested products were subsequently analyzed electrophorically; M*bo*II produced 277, 119 and 110 bp fragments in G6PD Mediterranean samples whereas 377 and 119 bp fragments were observed in normal subjects. DNA samples containing C at position 1311 showed 23 and 203 bp fragments with B*cl*I digestion whereas replacement of C by T at this nucleotide position resulted in a 180 bp product.

Undigested PCR products were sent to Macrogen^® ^(Seoul, Korea) for gene sequencing.

The frequency of 1311 polymorphism was evaluated and compared in two groups; (a) patients carrying G6PD Mediterranean gene (b) healthy individuals.

### Statistical Analysis

All calculations were computed using statistical package SPSS version 16.0 (SPSS inc.; Chicago, IL, USA). Pearson's chi-square test was used for the comparison of statistical difference between the groups and the threshold of significance (*p*-value) was set at 0.05.

### Ethical issues

The study was approved by institutional ethical review committee and informed consent was obtained from all subjects prior to their enrollment.

## Results

**Figure 1:**
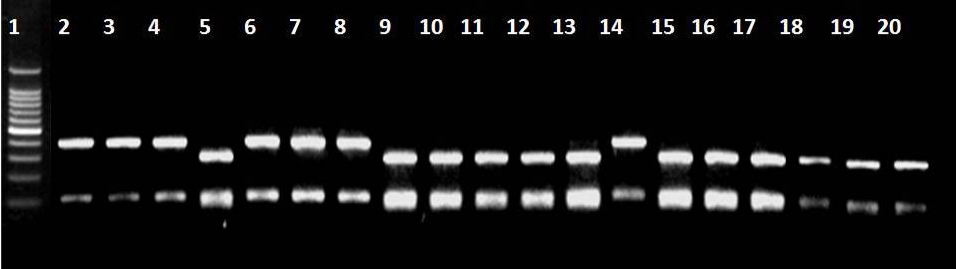
**Results of RFLP-PCR with *Mbo- *II digestion in G6PD deficient subjects**. Lane 1: 100 bp size marker; lanes: 5, 9–13, 15–20: 563C-T mutation.

**Figure 2:**
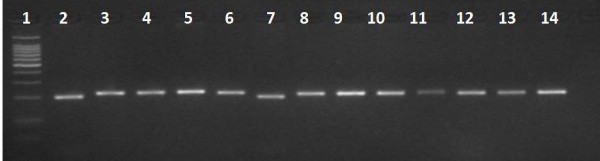
**Results of RFLP-PCR with B*cl*-I digestion in G6PD deficient individuals**. Lane 1:100 bp size marker; lane2, 7: 1311T polymorphism.

Two hundred and seventy four G6PD deficient subjects including 235 males and 39 females agreed to participate in the study. Of them 563 C-T mutation was observed in 187 males (79.6%) and in 27 females (69.2%). Thus, cumulative gene frequency for G6PD Mediterranean was 0.74 in both sexes detected in 234 of 313 X-chromosomes. Associated 1311 T allele gene frequency was 60/234 or 0.26. Gene sequencing was performed on 21 G6PD deficient DNA samples (three with 1311T and 18 with 1311C) which showed additional 93T/C polymorphism in intron 11: seven had 93 C and 14 had 93 T. Only two cases of combined mutations of 1311C-T and 93 T-C were observed. Both were males and their ages were 4 and 8 days at the time of diagnosis. Their associated G6PD mutation, if any remained undetected. RFLP-PCR results for 563 C-T and 1311 C/T are shown in figures 1 &2 and demographics and gender based results are detailed in Table 1.

**Table 1:** Analysis of X-chromosomes from G6PD deficient subjects with 563 C-T mutation (n = 214).

Gender	n	Age (years) mean ± SD	X chromosomes n (%)	1311T allele n (%)*	P-value
Males	187	16.8 ± 16.6	187(0.79)	48(0.26)	0.988
Females	27	8.2 ± 12.7	47** (0.60)	12(0.25)	
Total	214	9.3 ± 13.4	234(0.74)	60(0.26)	

* % of T allele is calculated in association with 563 C-T mutations only

** There were 20 homozygous and 7 heterozygous in this population group

The X-chromosomes of 400 unrelated subjects belonging to five major and 11 minor ethnicities were analyzed for 1311C/T polymorphism of G6PD gene. All except two were males with age ranging from 17 to 52 years (median ± SD 27.0 ± 7.0). G6PD levels of the participants were not performed. It was assumed based on clinical history that the enrolled subjects were not suffering from G6PD deficiency. Moreover, frequency of G6PD deficiency noted in general population of the country is 1.8–8.6% [22-30]. There fore, it was expected that the results would not be skewed even if deficient samples were included in the study. The distribution of 1311 T with respect to various ethnicities in Pakistan is summarized in Table 2 which demonstrated striking similarities among various ethnic groups living in Pakistan. It was observed that *the *polymorphism was frequent with an incidence of 0.28–0.38 in five main ethnic groups. Highest prevalence was observed in Saraiki subjects with a frequency of 0.5. In contrast, no polymorphism was observed in local Hindu population.

**Table 2:** Demographic details and gene frequency for 1311 C/T polymorphism of healthy subjects with respect to their ethnicity (n = 400, M: 398, F: 2)

**Ethnicity**	**n (%)**	**No. of X Chromosomes**	**1311C** **n (gene frequency)**	**1311T** **N (gene frequency)**
Mohajir*	68(17.0)	69	50(0.72)	19(0.28)
Pathan	61(15.3)	61	38(0.62)	23(0.38)
Punjabi	60(15.1)	60	41(0.68)	19(0.32)
Baloch	60(15.1)	60	44(0.73)	16(0.27)
Sindhi	60(15.1)	60	37(0.62)	23(0.38)
Gilgiti	17(4.3)	17	11(0.65)	6(0.29)
Sariki	14(3.5)	14	7(0.50)	7(0.50)
Gujrati*	11(2.8)	12	8(0.66)	4 (0.33)
Bhori	9(2.3)	9	6(0.67)	3(0.33)
Hindu	7(1.8)	7	7(1.00)	0(0.00)
Parsi	4(1.0)	4	3((0.75)	1(0.25)
Bangali	1(0.3)	1	0(0.00)	1(1.00)
Burhani	1(0.3)	1	0(0.00)	1(1.00)
Kashmiri	1(0.3)	1	0(0.00)	1(1.00)
Total	400(100)	402	272(0.67)	130(0.32)

* One female subject each in respective ethnic group

No significant statistical differences were assessed in 1311 T polymorphism in compared groups: Pathans and Jews (*p*-value 0.29), Mohajirs and Indians (*p*-value 0.32) and Baluchis and Iranians (*p*-value 0.71).

## Discussion

We performed an extensive analysis of 1311 C/T polymorphism of G6PD gene within normal Pakistani population and in subjects with G6PD deficiency. It was found that this polymorphism is quite frequent in normal and individuals with G6PD Mediterranean gene. The work enabled us to understand the genetic basis of G6PD deficiency in Pakistani population and to compare T allele frequency with various world populations including those that are considered traditionally to be origin or source of various ethnic groups residing in Pakistan.

Pakistan is a multilingual and multiethnic nation. Its various ethnic groups are unequally distributed through out the country. Punjabis and Pathans which are mainly located in the North Pakistan constitute 60% and 13% of the population respectively [31] while Sindhis and Baluchis occupy Southern Pakistan. There is considerable ethnic admixture among Punjabis because most of the incoming invaders in the past 2000 years passed through Punjab and mixed with previous settlers. In contrast, Pathans are claimed to be descendants of European soldiers who accompanied Alexander the Great [32] and are also considered to be the lost tribe of Jews [33]. Sindhis and Baluchis that are main occupants of Southern Pakistan comprise respectively of 13 and 8% of Pakistani population[34,35]. While former have mixed ethnic background, the Baluchis are believed to have originated in Aleppo (Syria) who migrated via Iran to this land [35]. Mohajirs constitute some 8% of population and are a group of people who migrated to Pakistan from near east – the present day India after its independence as a separate state in 1947 [35]. They lack the tribal based ethnic identity in contrast to the rest of Pakistanis.

### Polymorphism of 1311C/T with and without 563C-T mutation in Pakistani G6PD deficient population

C-T transition at NT 1311 was detected in 60/234 X-chromosomes carrying G6PD Mediterranean gene (equaling a gene frequency of 0.26). The frequency of this polymorphism was not statistically different for patients whose DNA did not manifest G6PD 563C-T as was detected in 11/72 X-chromosomes (0.15 gene frequency; p-value 0.189). A lower incidence of 0.13 was observed by Saha et al in 1994 for this silent mutation among subjects with G6PD Mediterranean in Pakistan[21].

### Association of 1311 C/T and 93 T/C polymorphisms

Recently, 1311 C/T polymorphism was observed in association with another silent mutation 93 T/C at intron 11 in South East Asians [36,37]and Chinese populations [38,39]. The association of two is significant in reducing G6PD enzyme activity and hence has clinical implications [39]. Unfortunately, gene sequencing results were available for limited number of G6PD deficient patients in the present study which showed a gene frequency of 0.66 for combined mutations of 1311C-T and 93 T-C.

### G6PD NT1311 Polymorphism in various ethnicities in Pakistan

G6PD NT 1311 C-T was observed uniformly in various ethnic groups in Pakistan with an overall frequency of 0.32. Different results seen in local Hindu and Sariki populations may be secondary to small sample size demanding further evaluation. A lower though not statistically significant frequency for T allele (0.20) was observed by Saha N et al in 1994 for Pashtoons and Punjabi population [21] in contrast to 0.3 in the present work for the same population (p-value 0.133).

### Comparison of NT 1311 polymorphism with global data

Global distribution of the silent mutation within normal population is summarized in Table 3. The reported frequency of 1311T is surprisingly high (0.45) among Asian Indians compared to other populations of the world [12]. Central and South America, Jews, Sicilians and Africans had considerably low gene frequency for this silent mutation ranging from 0.1 to 0.25 [12]. Beutler and Kuhl in 1990 reported frequency of 0.05 for the same in Orientals [12]. Recently the haplotype is recognized with increasing frequency in Chinese population [38,40] but the same is not true for Burmese and Mons [36]. Variable frequencies have been reported from Bahrain [41] Saudi Arabia [13] and Iran [42] as 0.30, 0.14 and 0.22 respectively. Similar to Americans, English (Caucasians) also showed a lower frequency of 0.137 for this polymorphism [42]. We found that haplotype1311T is prevalent extensively in our various ethnic groups with the overall frequency of 0.32 in general population.

**Table 3:** Global Distribution of T allele gene in various populations

Population	No of X-chromosomes studied	1311 T gene frequency	Reference
African	20	0.25	[12]
White Jewish	41	0.220	[12]
White Non-Jewish	68	0.132	[12]
Sicilian	18	0.167	[12]
English (Caucasians)	436	0.137	[42]
Central/South American	30	0.100	[12]
Indian	20	0.45	[12]
Chinese	23	0.34	[40]
Burmese	17	0.05	[36]
Mons	19	0.00	[36]
Saudi Arabian	14	0.143	[13]
Iranian	100	0.22	[42]
Bahraini	117	0.30	[41]
Pakistani	402	0.32	Present study

### Assessment of 1311 polymorphism with Source Population

Qamar R et al in 2002 through their extensive work on Y-chromosomal DNA supported origin of Parsis in Iran, Hazaras from Genghis Khan's army and Negroid Makrani in Africa [43]. However, they denied the Syrian, Tibetan, Greek or Jewish origin for Baluch, Balti and Pathan population respectively. Subsequent work revealed limited Greek contribution to Pakistani Pathan Population [32]. Our work was limited in investigating a single allele which by no means allows us to compare the populations. Also, statistical comparison between two unrelated studies can not be conclusive. However, it was interesting to note that Pathans and Jews, Mohajir and Indians and Baluch and Irani revealed insignificant statistical differences in 1311 T frequency. Other groups like Punjabis and Sindhis were not compared with any population because of their 'mix' origin.

In this study, we report the presence of silent mutation in normal G6PD alleles in a frequency of 0.32 which apart from Asian Indians [12] is highest reported from any population.

## Conclusion

We conclude that G6PD nucleotide 1311 T polymorphism is relatively common in Pakistani population both with and without G6PD Mediterranean. It is expected that the work will help in understanding genetic basis of G6PD deficiency in Pakistani population and establishing ancestral links between various ethnicities residing in this region.

## Authors' contributions

BM designed the study, analyzed the data and wrote the paper. AN performed bench work, analyze the results and wrote part of paper. TM wrote part of paper and critically evaluated the manuscript. ZAN did bench work and analyzed the results. MK critically evaluated the paper. All authors approved final form of manuscript before submission.

## References

[B1] Beutler E (1990). The genetics of glucose-6-phosphate dehydrogenase deficiency. Semin Hematol.

[B2] Pai GS, Sprenkle JA, Do TT, Mareni CE, Migeon BR (1980). Localization of loci for hypoxanthine phosphoribosyltransferase and glucose-6-phosphate dehydrogenase and biochemical evidence of nonrandom X chromosome expression from studies of a human X-autosome translocation. Proc Natl Acad Sci USA.

[B3] Beutler E, Vulliamy TJ (2002). Hematologically important mutations: glucose-6-phosphate dehydrogenase. Blood Cells Mol Dis.

[B4] Beutler E (1994). G6PD deficiency. Blood.

[B5] Linder D, Gartler SM (1965). Glucose-6-phosphate dehydrogenase mosaicism: utilization as a cell marker in the study of leiomyomas. Science.

[B6] Beutler E, Collins Z, Irwin LE (1967). Value of genetic variants of glucose-6-phosphate dehydrogenase in tracing the origin of malignant tumors. N Engl J Med.

[B7] Fialkow PJ (1976). Clonal origin of human tumors. Biochim Biophys Acta.

[B8] Pack SD, Kolonin MG, Borodin PM, Searle JB, Serov OL (1995). Gene mapping in the common shrew (Sorex araneus; Insectivora) by shrew-rodent cell hybrids: chromosome localization of the loci for HPRT, TK, LDHA, MDH1, G6PD, PGD, and ADA. Mamm Genome.

[B9] De bruyn A, Raeymaekers P, Mendelbaum K, Sandkuijl LA, Raes G, Delvenne V, Hirsch D, Staner L, Mendlewicz J, Van Broeckhoven C (1994). Linkage analysis of bipolar illness with X-chromosome DNA markers: a susceptibility gene in Xq27-q28 cannot be excluded. Am J Med Genet.

[B10] Fey MF, Wainscoat JS, Mukwala EC, Falusi AG, Vulliamy TJ, Luzzatto L (1990). A PvuII restriction fragment length polymorphism of the glucose-6-phosphate dehydrogenase gene is an African-specific marker. Hum Genet.

[B11] Vulliamy TJ, Othman A, Town M, Nathwani A, Falusi AG, Mason PJ, Luzzatto L (1991). Polymorphic sites in the African population detected by sequence analysis of the glucose-6-phosphate dehydrogenase gene outline the evolution of the variants A and A. Proc Natl Acad Sci USA.

[B12] Beutler E, Kuhl W (1990). The NT 1311 polymorphism of G6PD: G6PD Mediterranean mutation may have originated independently in Europe and Asia. Am J Hum Genet.

[B13] Kurdi-Haidar B, Mason PJ, Berrebi A, Ankra-Badu G, al-Ali A, Oppenheim A, Luzzatto L (1990). Origin and spread of the glucose-6-phosphate dehydrogenase variant (G6PD-Mediterranean) in the Middle East. Am J Hum Genet.

[B14] Newcomb L (1986). The Islamic Republic of Pakistan: country profile. Int Demogr.

[B15] Bernhard W (1983). Ethnogenesis of South Asia with special reference to India. Anthropol Anz.

[B16] Birdwood (1959). Review: A History of the Pathans: Review. The Geographical Journal.

[B17] Lapidus IM (2002). A history of Islamic societies.

[B18] Black C (2003). Pakistan. The people.

[B19] Hussain R, Bittles AH (1998). The prevalence and demographic characteristics of consanguineous marriages in Pakistan. Journal of biosocial science.

[B20] Wahab A, Ahmad M (1996). Biosocial perspective of consanguineous marriages in rural and urban Swat, Pakistan. Journal of biosocial science.

[B21] Saha N, Ramzan M, Tay JS, Low PS, Basair JB, Khan FM (1994). Molecular characterisation of red cell glucose-6-phosphate dehydrogenase deficiency in north-west Pakistan. Hum Hered.

[B22] Ronald AR, Underwood BA, Woodward TE (1968). Glucose-6-phosphate dehydrogenase deficiency in Pakistani males. Trans R Soc Trop Med Hyg.

[B23] McCurdy PR, Mahmood L (1970). Red cell glucose-6-phosphate dehydrogenase deficiency in Pakistan. J Lab Clin Med.

[B24] Hashmi JA, Farzana F, Ahmed M (1976). Abnormal hemoglobins, thalasemia trait & G6PD deficiency in young Pakistani males. J Pak Med Assoc.

[B25] Bollinger RC (1985). Glucose 6 phosphate dehyrogenase deficiency in Lahore area. Pak J Med Res.

[B26] Khattak MFDM, Saleem M (1992). The prevalence of glucose 6 phosphate dehydrogenase deficiency in Nortehrn Pakisatn. Pak Armed Forces MEd J.

[B27] Bouma MJ, Goris M, Akhtar T, Khan N, Khan N, Kita E (1995). Prevalence and clinical presentation of glucose-6-phosphate dehydrogenase deficiency in Pakistani Pathan and Afghan refugee communities in Pakistan; implications for the use of primaquine in regional malaria control programmes. Trans R Soc Trop Med Hyg.

[B28] Khan M (2004). Glucose 6 phosphate dehydrogenase deficiency in adults. J Coll Physicians Surg Pak.

[B29] Khan TAAS, Anwar M, Ayub M (2004). The frequancy of glucose 6 phosphate deficeint in Punjabis and Pathans. J Post Grad Med Inst.

[B30] Ali N, Anwar M, Ayyub M, Bhatti FA, Nadeem M, Nadeem A (2005). Frequency of glucose-6-phosphate dehydrogenase deficiency in some ethnic groups of Pakistan. J Coll Physicians Surg Pak.

[B31] Wirsing RG (1987). The Baluchis and Pathans. London.

[B32] Firasat S, Khaliq S, Mohyuddin A, Papaioannou M, Tyler-Smith C, Underhill PA, Ayub Q (2007). Y-chromosomal evidence for a limited Greek contribution to the Pathan population of Pakistan. Eur J Hum Genet.

[B33] Papiha SS, Deka R, Chakraborty R (1999). Genomic diversity: applications in human population genetics.

[B34] Wilber DN (1966). The nations of Asia.

[B35] Rengel M (2003). Pakistan: a primary source cultural guide.

[B36] Nuchprayoon I, Louicharoen C, Charoenvej W (2008). Glucose-6-phosphate dehydrogenase mutations in Mon and Burmese of southern Myanmar. Journal of human genetics.

[B37] Matsuoka H, Thuan DT, van Thien H, Kanbe T, Jalloh A, Hirai M, Arai M, Dung NT, Kawamoto F (2007). Seven different glucose-6-phosphate dehydrogenase variants including a new variant distributed in Lam Dong Province in southern Vietnam. Acta medica Okayama.

[B38] Yu GL, Jiang WY, Du CS, Chen LM, Lin QD, Tian QH, Zeng JB, Li SG (2004). [Identification of G6PD gene variants from Hakka population in Guangdong province]. Zhonghua yi xue yi chuan xue za zhi = Zhonghua yixue yichuanxue zazhi = Chinese journal of medical genetics.

[B39] Yu GL, Jiang WY, Du CS, Lin QD, Chen LM, Tian QH, Li SG, Zeng JB (2004). [Complex mutations of 1311 C-->T in exon 11 and 93 T-->C in intron 11 in G6PD gene]. Zhonghua xue ye xue za zhi = Zhonghua xueyexue zazhi.

[B40] Yang Z, Chu J, Ban G, Huang X, Xu S, Li M (2001). [The genotype analysis of glucose-6-phosphate dehydrogenase deficiency in Yunnan province]. Zhonghua yi xue yi chuan xue za zhi = Zhonghua yixue yichuanxue zazhi = Chinese journal of medical genetics.

[B41] Nabeel Al Momen SSAA, Ahmed Al Alawi A (2004). Molecular Homogenity of G6PD deficiency. Bahrain Medical Bulletin.

[B42] Mortazavi Y, Chopra R, Gordon-Smith EC, Rutherford TR (1997). Frequency of the G6PD nt 1311 C/T polymorphism in English and Iranian populations: relevance to studies of X chromosome inactivation. J Med Genet.

[B43] Qamar R, Ayub Q, Mohyuddin A, Helgason A, Mazhar K, Mansoor A, Zerjal T, Tyler-Smith C, Mehdi SQ (2002). Y-chromosomal DNA variation in Pakistan. Am J Hum Genet.

